# Evolution Characteristics of Seismic Detection Probability in Underground Mines and Its Application for Assessing Seismic Risks—A Case Study

**DOI:** 10.3390/s22103682

**Published:** 2022-05-12

**Authors:** Hui Li, Anye Cao, Siyuan Gong, Changbin Wang, Rupei Zhang

**Affiliations:** 1 School of Mines, China University of Mining and Technology, Xuzhou 221116, China; lihui@cumt.edu.cn (H.L.); gsy_cumt@cumt.edu.cn (S.G.); zrp@cumt.edu.cn (R.Z.); 2 Jiangsu Engineering Laboratory of Mine Earthquake Monitoring and Prevention, China University of Mining and Technology, Xuzhou 221116, China; 3 State Key Laboratory of Coal Resources and Safe Mining, China University of Mining and Technology, Xuzhou 221116, China; changbin.wang@cumt.edu.cn

**Keywords:** coal burst, rockbursts, detection probability, seismic monitoring, magnitude of completeness, seismic data integrity

## Abstract

Seismic hazards are typical mining hazards causing dynamic failure of coal and rock mass, which greatly threatens the safety of personnel and equipment. At present, various seismic analysis methods are used to assess seismic risks but their accuracy is significantly limited by the incompleteness of seismic data. The probability of detecting earthquakes (*PDE*) method has been proven as a powerful means for retrieving missed seismic events and enhancing the seismic data integrity in mines. However, to date, the reliability of the results of the *PDE* method has not been assessed and the highly integrated seismic data have not been linked with the actual hazard potential. To fill these gaps, this paper investigated the impacts of the seismic data volume used for calculation and the modification of the layout of sensors on the reliability and robustness of the *PDE* method. The event counts and seismic energy were compensated using the *PDE* method, correlated with strong seismic events. The results indicated that the compensated seismic data presented higher accuracy in locating future hazardous events than before. This research provides references on enhancing the performance of seismic analysing methods for seismic risk assessments.

## 1. Introduction

Seismic hazards such as coal burst and rockbursts are the most formidable mining hazards in underground mines. They describe the unexpected dynamic failure of coal or rock mass around mine openings, accompanied with a sudden release of strain energy and violent material ejections [1,2]. In recent decades, because of the high in-situ stress in deep mines and the challenging underground environment, seismic hazards have occurred frequently in most mining countries, including China, Australia, the US, and Poland [1,3,4,5]. To ensure the safety of personnel and equipment in mines, methods must urgently be developed that can provide efficient seismic risks evaluation and dynamic failure prediction prior to hazard occurrence.

The routine seismic monitoring technology in mines plays an important role in the prediction and control of seismic hazards. By detecting seismic waves emitted from rock fractures, seismic monitoring provides a powerful means of locating internal damage in the coal and rock mass by investigating the source mechanism [6]. Based on various source parameters, many seismic analysing methods for evaluating seismic risks have been developed. Prominent examples are the variation of event counts and seismic energy [7,8,9,10,11], seismic clustering [12,13], wave velocity tomography [14,15,16], and ground motion analysis [17,18,19,20], which obtained encouraging results in seismic hazard forecasting. However, because of the complicated monitoring environment, the performance of seismic analysing methods can be significantly limited by poor seismic data integrity. Seismic data integrity describes the completeness degree of seismic recordings that the system can monitor from the coal and rock mass. Limited by the layout of excavations, seismometers cannot fully cover the whole area of interest [21]. For the area not enclosed by seismometers, it is more likely that medium-low energy events are missed by the seismic monitoring system, as their seismic waves are strongly attenuated and fail to be detected, due to its low signal to noise ratio that is under the sensibility of a common seismometer. Not considering the incompleteness of seismic data may induce biases in the seismic analysis results, which significantly lowers the accuracy in assessing seismic risks [22].

In seismology, the integrity of the earthquake catalogue is commonly evaluated by the magnitude of completeness *m_c_* [23]. *m_c_* is defined as the minimum magnitude of events the seismic monitoring system can fully detect [24,25,26]. However, because of the sparse seismic data characteristics and human-made noises in underground mines, it is difficult to apply *m_c_* for evaluating the integrity of seismic data during the mining process. A novel method based on the probability of detecting earthquakes, hereafter called the *PDE* method, was proposed by Schorlemmer and Woessner [27]. This method assesses the seismic data integrity in terms of the probability of the seismic monitoring system to detect seismic events. As the *PDE* method is not constrained by sparse data and can tolerate various source types, and it is ideally suited to evaluate seismic data integrity in the mining environment. Detailed research on the application of the *PDE* method in mines was conducted by Wang et al. [22], who investigated the seismic detection characteristics of individual geophones and seismic monitoring systems for events with different energies. The results indicated that the *PDE* method potentiality retrieved missing seismic events and provided insights into correcting the seismic analysing results for seismic risk evaluation. However, further investigations on the *PDE* method are still required before it can be applied to other seismic analysing methods. As the *PDE* method is based on historical seismic recordings, the reliability of the detection probability results is directly controlled by the amount of historical seismic data used for calculation. Therefore, determining an optimal historical seismic data volume that can maximise the result reliability and robustness and minimise the data collection time is essential. Apart from that, during longwall mining, the locations of geophones in roadways are regularly modified when the longwall face is nearby. According to the *PDE* method, the detection capacity of a geophone should be recalculated after it has been moved, which will influence the overall detection probability of the seismic monitoring system. To maintain the robustness of the results, the detection probability characteristics before and after the movement of geophones should be investigated. Additionally, there is still no relevant research on the correlation between highly integrated seismic data and seismic risks.

Therefore, in this paper, the evolution characteristics of the detection probability of the seismic monitoring system during the longwall retreat were investigated, and highly integrated seismic data were further correlated with seismic risks. This study is based on threemonths of seismic data in a burst-prone coal mine. The evolution characteristics of the detection capacity of individual geophones after the commencement of seismic monitoring were investigated first. The amount of seismic data and the time required for data collection for deriving reliable detection capacity results were determined. Then, the evolution characteristics of the detection probability of the seismic monitoring system before and after a geophone movement were studied, and the reliability and robustness of the detection probability results were discussed. Based on the results of the detection probability, raw seismic data were compensated to retrieve the actual seismic activities that have occurred in the coal and rock mass. To assess the seismic hazard potential, the event counts and seismic energy were correlated with the hazardous seismic eventsusing compensated seismic data. The results of this research provide insights into how to enhance the performance of seismic analysing methods for assessing seismic risks in mines.

## 2. Methodology

### 2.1. Probability of Detecting Earthquakes Method

**Figure 1:**
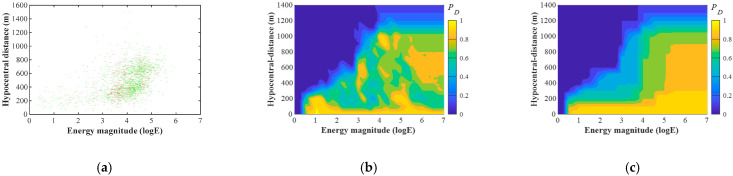
Examples of (**a**) a distribution of picked (green dots) and unpicked (red dots) seismic events of a geophone in the D-M map, (**b**) $P_{D}$ result of a geophone before applying constraints, and (**c**) $P_{D}$ result of a geophone after applying constraints [22].

This section briefly introduces the probability of detecting earthquakes (*PDE*) method for evaluating the detection capacity of geophones and the detection probability of seismic monitoring systems in mines. More details can be found in Wang et al. [22]. In this study, the energy of seismic events is displayed in the base-10 logarithm of the seismic energy (*logE*). For example, a 100 kJ seismic event has a *logE* of 5. According to the classical locating algorithm using the least-square method, a seismic event can be identified by the onset time of its seismic waves recorded by at least four geophones [28]. Because a seismic wave is attenuated by the increased distance between the geophone and the seismic source (hereafter referred to as hypocentral distance), it is easier for geophones to pick the onset time of seismic waves if the seismic source has a higher energy and/or a lower hypocentral distance. Inversely, geophones may receive a blurry seismic wave from a seismic source with a lower energy or/and a higher hypocentral distance, making it difficult to accurately pick the onset time. Therefore, the capacity of a geophone to pick seismic waves, i.e., the detection capacity ($P_{D}$), can be represented by plotting both the picked and unpicked seismic events in the hypocentral distance-energy (D-M) coordinate, which is called the D-M map. A typical example of a D-M map is shown in Figure 1a, where picked and unpicked seismic events are represented by green and red dots, respectively. Based on the D-M map, the $P_{D}$ of the geophone for an upcoming seismic event with energy and hypocentral distance R can be calculated as [22]:

(1) $$P_{D}\left(logE,R\right)=\{\begin{array}{c} \frac{N_{+}}{N_{+}+N_{-}}\;\;\;\;\;\;\;\left(N_{+}+N_{-}\geq N_{min}\right)\\ \frac{N_{+}}{N_{min}}\;\;\;\;\;\;\;\;\;\;\;(N_{+}+N_{-}<N_{min}) \end{array}$$

where $N_{+}$ and $N_{-}$ are the number of picked and unpicked historical seismic events around an upcoming seismic event. $N_{min}$ is the minimum number of events required for the $P_{D}$ calculation, which is the key parameter to control the robustness and effectiveness of the $P_{D\;}$ results. The details of determining $N_{min}$ can be found in Wang [29], and $N_{min}$ = 9 was adopted in the studied longwall. By gridding the D-M map, the $P_{D}$ of the geophone for the seismic events with different energies and hypocentral distances can be derived, as shown in Figure 1b. However, because of the sparse seismic data characteristics in the D-M map, a counterintuitive $P_{D}$ distribution in Figure 1b is commonly observed. In this distribution, $P_{D}$ for seismic events with higher energies and/or lower hypocentral distance can be lower than events with lower energies and/or higher hypocentral distance. Therefore, two constraints are further applied to correct the $P_{D}$ result: (1) with the same hypocentral distance, $P_{D}$ for a higher energy cannot be lower than that for a lower energy; (2) with the same energy level, $P_{D}$ for a lower hypocentral distance cannot be higher than that for a higher hypocentral distance. Figure 1c shows the $P_{D}$ distribution result after applying constraints.

Based on the $P_{D}$ results of the available geophones, the probability that the seismic monitoring system detects seismic events in the area of interest can be derived. Assuming that a total of *m* geophones are available in the seismic monitoring system, the probability of a seismic event detected by *n* geophones can be calculated by multiplying $P_{D}$ of these *n* geophones and $1-P_{D}$ of the remaining *m* − *n* geophones. As there are $C_{m}^{n}$ combinations of *n* geophones, the detection probability for *n* geophones to detect a seismic event, $P_{E}^{n}$, is calculated as [22]:

(2) $$P_{E}^{n}=\overset{C_{m}^{n}}{\underset{k=1}{\sum }}P_{E}^{n\left(k\right)}$$

where *k* is the *k-*th combination of geophones, which ranges from 1 to $C_{m}^{n}$.

Identifying a seismic event requires at least four geophones that have picked the onset time of the seismic waves, which also means that a seismic event cannot be recorded if its seismic waves are only picked by no more than three geophones. Therefore, for a seismic event at location (x,y) with energy logE, the probability of it being detected by the seismic monitoring system is [22]:

(3) $$P_{E}\left(x,y,logE\right)=1-\overset{3}{\underset{n=0}{\sum }}P_{E}^{n}$$

### 2.2. Compensated Seismic Data

According to Section 2.1, $P_{E}$ is the probability for the seismic monitoring system to detect a seismic event with a given hypocentral distance and energy. This also means that there is a probability of $1-P_{E}$ to miss such a seismic event. The detection probability of medium-low energy events located in the area where geophones are not sufficiently enclosed is potentially at a lower level, which may lead to inaccurate seismic analysis results of event counts and seismic energy. Therefore, it is ideal to compensate raw seismic recordings that can represent the actual seismic responses towards mining activities. For a given area where *m* seismic events have occurred, compensated event counts ($Num_{com}$) and energies ($\log E_{com}$) are calculated as:

(4) $$Num_{com}=1/P_{E}\left(x_{1},y_{1},logE_{1}\right)+1/P_{E}\left(x_{2},y_{2},logE_{2}\right)\cdots +1/\;P_{E}\left(x_{m},y_{m},logE_{m}\right)$$

(5) $$\log E_{com}=log[E_{1}/P_{E}\left(x_{1},y_{1},logE_{1}\right)+E_{2}/P_{E}\left(x_{2},y_{2},logE_{2}\right)\cdots +E_{m}/\;P_{E}\left(x_{m},y_{m},logE_{m}\right)]$$

where $E_{1},\;E_{2},E_{m}\ldots$ are the seismic energy levels of events, and $P_{E}\left(x_{1},y_{1},logE_{1}\right),\;P_{E}\left(x_{2},y_{2},logE_{2}\right),\;P_{E}\left(x_{m},y_{m},logE_{m}\right)\ldots$ are the detection probabilities of *m* events in the area of interest.

### 2.3. Detection Probability Similarity

Based on the *PDE* method, Section 2.2 provides a practical way to compensate raw seismic data for the analyses. However, as the *PDE* method uses historical seismic recordings, the detection capacity of geophones ($P_{D}$) and the detection probability of the seismic monitoring system ($P_{E}$) can only be calculated after a period of raw seismic data collection. More reliable and robust $P_{D}$ and $P_{E}$ results can be expected if more seismic data are available, but more time is also consumed before they can be applied to compensate for raw seismic data. It can be assumed that the last $P_{D}$ and $P_{E}$ results before each modification of the geophones layout, named $P_{D\left(last\right)}$ and $P_{E\left(last\right)}$, commonly have the highest reliability and robustness as they use the most seismic data for calculation. Therefore, for a given geophone layout, the reliability and robustness of the $P_{D}$ and $P_{E}$ results at different periods can be assessed by calculating their similarity with $P_{D\left(last\right)}$ and $P_{E\left(last\right)}$, respectively. In this study, $P_{D}$ and $P_{E}$ similarity was calculated using the cosine similarity method [30]. This method calculates the cosine of the angle between two data arrays. The smaller this angle, the higher the similarity between two data arrays. The cosine of the angle between the arrays $\vec{A}$ and $\vec{B}$ is calculated as:

(6) $$\cos\theta =\frac{\vec{A}\cdot \vec{B}}{\left\|A\|\|B\right\|}=\frac{\sum_{i=1}^{n}A_{i}B_{i}}{\sqrt{\sum_{i=1}^{n}A_{i}{}^{2}}\sqrt{\sum_{i=1}^{n}B_{i}{}^{2}}}$$

## 3. Site Overview

### 3.1. Geological and Mining Conditions

**Figure 2:**
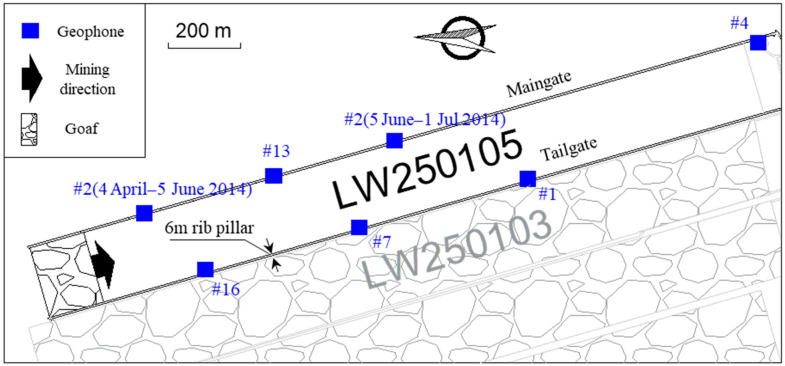
LW250105 layout and geophone distribution in roadways. The location of Geophone #2 was modified on 5 June 2014.

The study was conducted in a burst-prone longwall, namely Longwall (LW) 250,105 in Huating Coal Mine, Gansu Province, China. The target coal seam has an average thickness of up to 40 m, with a depth of 550–800 m and a dip angle ranging between 1°–15°. The seam is overlain by 15 m of sandy mudstone and 25 m of sandstone layers, and it is underlain by 2 m of sandy mudstone and 15 m of sandstone layers. Because of the extreme thickness, the target coal seam was extracted by using the multi-slice mining method, where the height of each slice was about 13 m. LW250105 was in the top slice of the coal seam, and the longwall top caving method was used for extraction, with a mining height of 5 m and a caving height of 8 m. The longwall is 2000 m long and 200 m wide, and it started to retreat in March 2014 and stopped in 2016. The west side of the longwall is the goaf zone of the previous longwalls in the top slice of the target coal seam. To reduce coal losses, LW250105 was developed by using the “gob-side entry driving” method [31], and only a 6-m-wide rib pillar was designed between the LW250105 tailgate and the goaf zone (see Figure 2). Therefore, during the longwall retreating period, hazardous seismic events and severe dynamic failure were frequently reported in the tailgate.

### 3.2. Seismic Monitoring System and Seismic Hazards

Before the retreat of LW250105, Huating coal mine installed an “SOS” seismic monitoring system from the Central Mining Institute of Poland. The system is equipped with 16 uniaxial geophones, including six in the LW250105 roadways and ten in excavations far away from the longwall. The geophones have a response of 1–600 Hz, a sampling rate of 500 Hz, a maximum data transmitting rate of 1 MB/s, and a 16-bit A/D conversion. In this study, seismic events that occurred from 5 April 2014 to 1 July 2014 in LW250105 were used for the analysis. During the study period, Geophones #1, #2, #7, #13, and #16 were available to monitor the seismic activities of the longwall. Additionally, Geophone #2 was moved on 5 June 2014 because of the approach of the longwall face, and the layout of geophones before and after the movement of Geophone #2 is shown in Figure 2. As illustrated in the Introduction, when using the *PDE* method, each movement of geophone(s) requires recalculations of $P_{D}$ of the moved geophone(s), which can influence the overall $P_{E}$ characteristics of the seismic monitoring system. Therefore, this study focused on the characteristics of the evolution of $P_{D}$ results of Geophone #2 before and after the movement, and then investigated its impact on the $P_{E}$ results of the seismic monitoring system.

**Figure 3:**
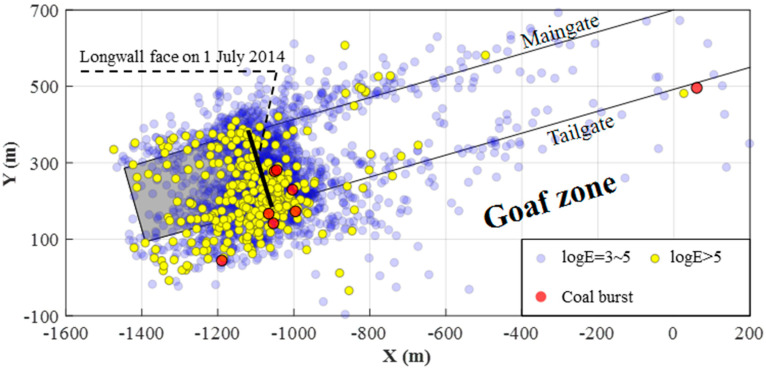
Distribution of coal bursts and seismic events with different energies in LW250105 from 5 April to 1 July 2014. The shaded zone denotes the active goaf behind the longwall face on 1 July 2014.

Because of the significant stress concentration in the coal and rock mass induced by the narrow rib pillar, LW250105 has experienced seven coal bursts and more than 300 strong seismic events with energy over 100 kJ during the study period. Figure 3 shows the distribution of coal bursts and seismic events with different energies in LW250105. Coal burst events gathered and caused severe damage in the tailgate. Many strong seismic events with *logE* > 5 also clustered on the tailgate side, which indicates a great pressure and stress concentration in the zone. Strong seismic events can be hazardous as their dynamic impacts can trigger dynamic failure in the highly stressed coal and rock mass. Therefore, seismic events with *logE* > 5 in LW250105 were used to investigate their correlation with the spatial event counts and seismic energy distribution using compensated seismic data.

## 4. Evolution of Detection Probabilities of the Seismic Monitoring System

### 4.1. Detection Capacity of Geophones (P_D_)

**Figure 4:**
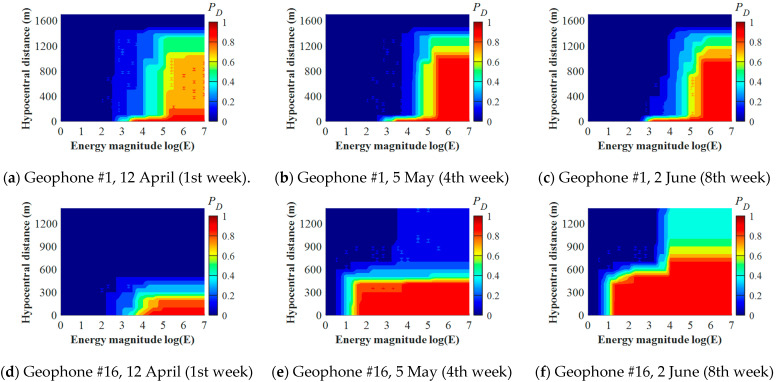
Evolution of Geophone #1 (**a**–**c**) and #16 (**d**–**f**) at different weeks.

Detection capacity ($P_{D}$) assessments for Geophones #1, #2, #7, #13, and #16 in LW250105 were conducted weekly during the study period. $P_{D}$ results of Geophone #1 and #16 in the 1st, 4th, and 8th week are used to illustrate the $P_{D}$ evolution characteristics, which are shown in Figure 4. According to Figure 4a–c, during all three weeks, Geophone #1 presented similar capacities for detecting seismic events with *logE* < 5. However, compared with the 1st week (Figure 4a), the $P_{D}$ results of Geophone #1 in the 4th and 8th week have a significant improvement for detecting seismic events with *logE* > 5 (Figure 4a,b). This demonstrates that Geophone #1 can update its $P_{D}$ results by picking seismic waves from events with *logE* > 5, whereas few seismic events can be detected with *logE* < 5. It can therefore be postulated that Geophone #1 was insensitive to seismic events with medium-low energy. Compared with Geophone #1, Geophone #16 presents significant differences of its $P_{D}$ results during the three weeks (see Figure 4d–f). In the 4th week, the $P_{D}$ results of Geophone #16 shown in Figure 4e present an improved detection capacity for events with *logE* < 4 than those in the 1st week shown in Figure 4d. Additionally, Geophone #16 had an enlarged detection range for seismic events with hypocentral distances of 400–600 m, where $P_{D}\;$> 0.5 is presented (see Figure 4e). In the 8th week, Geophone #16 had a further strengthened detection capacity for events with hypocentral distances of 600–900 m (see Figure 4f). This demonstrates that Geophone #16 is more sensitive to seismic events than Geophone #1 because of its higher detection ranges for seismic energy and hypocentral distances.

**Figure 5:**
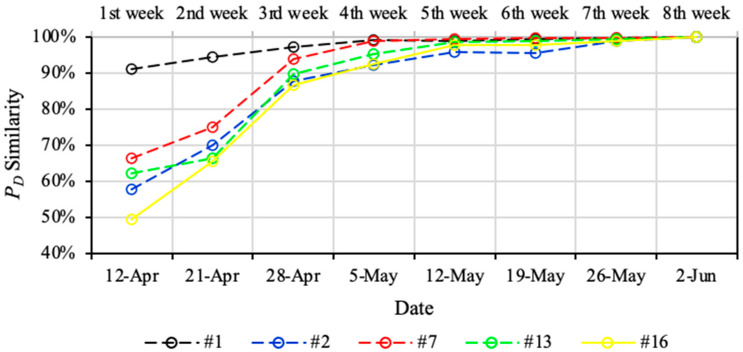
Similarity variation of geophones in LW250105 from 12 April to 2 June 2014. The similarity results of each geophone are calculated by comparing the final $P_{D}$ results on 2 June 2014.

According to Section 2.3, $P_{D\left(last\right)}$ of a geophone before relocation uses the most seismic recordings for $P_{D}$ calculation, and it is regarded as having the highest reliability. Therefore, using Equation (6), the reliability of the $P_{D}$ result of a geophone can be assessed by its similarity with its $P_{D\left(last\right)}$. Figure 5 shows the $P_{D}$ similarities of the five available geophones in LW250105 for 12 April to 2 June 2014. For each geophone, the $P_{D}$ similarity for a given time period is calculated by comparing it with the $P_{D}$ result on 2 June 2014, which is the $P_{D\left(last\right)}$ before the geophone movement on 5 June 2014. Figure 5 indicates significant differences of $P_{D}$ similarities over the first three weeks between different geophones. On 12 April 2014, Geophone #1 had the best $P_{D}$ results of the first week, which presented 91% similarity. In contrast, the $P_{D}$ similarities of the other four geophones were much lower with values ranging from 49% to 66%. The reason for this phenomenon may be the insensitivity of Geophone #1 for detecting medium-low energy seismic events. The number of high-energy seismic events is much lower than that of medium-low energy seismic events, which results in similar $P_{D}$ results of Geophone #1 in the high energy zone in the D-M map. However, from the 2nd week, Geophones #2, #7, #13, and #16 showed a dramatic increase of their $P_{D}$ similarities, which were all more than 90% in the 4th week. After the 4th week, only a tiny increase of $P_{D}$ similarities was present in all five geophones in the following weeks, which were between 90% and 100%. This implies that robust $P_{D}$ results for the geophones in LW250105 can be derived by collecting at least four weeks of seismic recordings.

**Figure 6:**
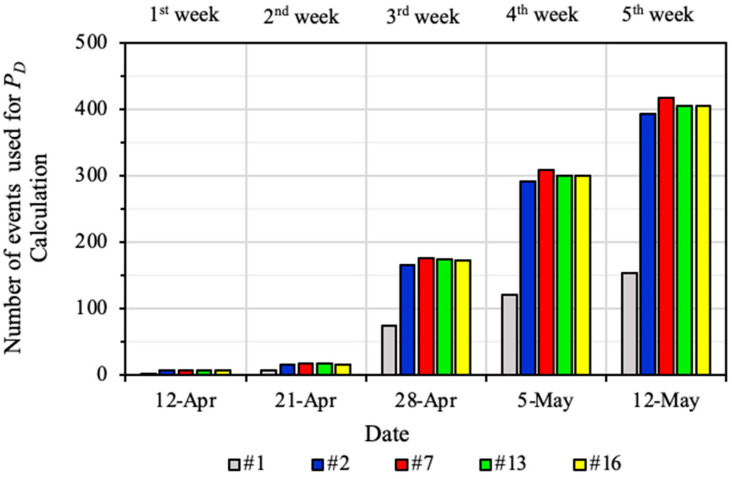
Number of seismic events used for weekly $P_{D}$ calculation for different geophones between 12 April and 12 May 2014.

Figure 6 shows the number of seismic events used for weekly $P_{D}$ calculation before their $P_{D}$ similarities exceeded 95%. As geophone #1 is only sensitive to high-energy events, it only presents less than half of the number of seismic events that other geophones used for $P_{D}$ calculation. For Geophones #2, #7, #13, and #16, about 300 seismic events were collected in the 4th week when their $P_{D}$ similarities exceeded 90%. This demonstrates that for the geophones in LW250105 that are sensitive to a wide range of seismic energy, collection of about 300 seismic events is required for deriving reliable and robust $P_{D}$ results.

**Figure 7:**
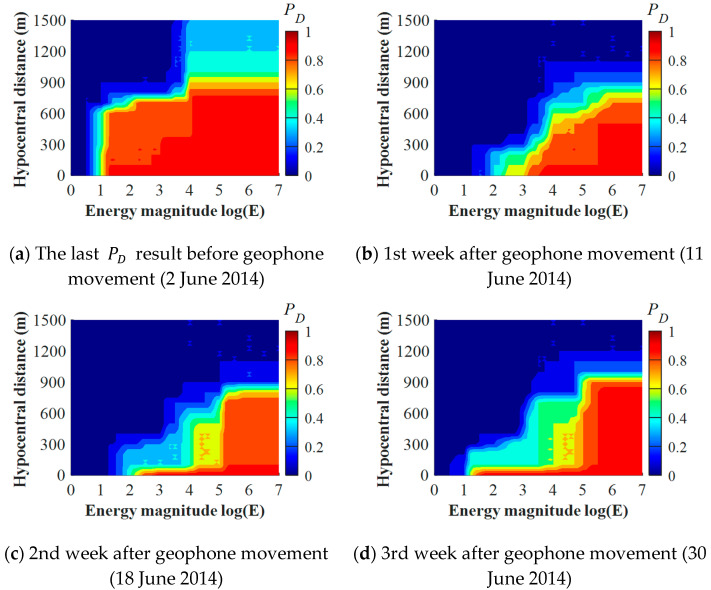
Detection capacity result of Geophone #2 before (**a**) and after (**b**–**d**) its movement on 5 July 2014 in LW250105.

To investigate the $P_{D}$ evolution characteristics over geophone movement, the $P_{D}$ results for Geophone #2 were calculated before and after its movement on 5 July 2014. The results are shown in Figure 7. For the last $P_{D}$ result before the movement (Figure 7a), Geophone #2 presented a significant sensitivity to seismic events with a wide energy range. For the 1st week after the movement (Figure 7b), Geophone #2 showed a decreasing detection capacity for detecting seismic events with *logE* < 3 and hypocentral distances larger than 300 m. During the 2nd and 3rd weeks (Figure 7c,d), although Geophone #2 presented an enhancing detection capacity in detecting high-energy events with *logE* > 5, it still retained a lower level of sensitivity for detecting seismic events with *logE* < 3, which is highly different from the $P_{D}$ result before geophone movement (Figure 7a). This demonstrates that the movement of geophones may significantly change the detection capacity characteristics.

### 4.2. Detection Probability of the Seismic Monitoring System (P_E_)

**Figure 8:**
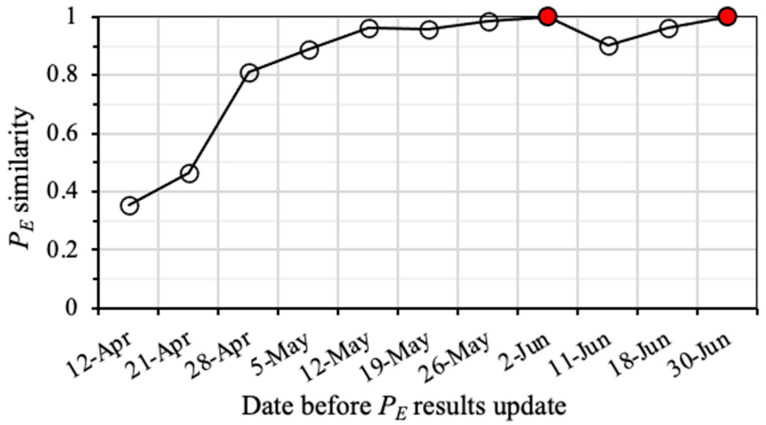
Similarity of weekly $P_{E}$ results for the seismic monitoring system to detect seismic events with *logE* = 4 in LW250105 during April to June 2014. Red dots are the last $P_{E}$ result before the layout modification of geophones.

Based on the $P_{D}$ results of the available geophones in LW250105 during the study period, the weekly $P_{E}$ distributions of seismic events with different energies were derived. Similar with the $P_{D}$ similarity calculation, $P_{E}$ similarities at different weeks were assessed by using the $P_{E\left(last\right)}$ result before the layout modification of each geophone. Figure 8 shows the $P_{E}$ similarity results for seismic events with *logE* = 4 in LW250105 from April to June 2014. $P_{E}$ results on June 2 and 30 June 2014 were the $P_{E\left(last\right)}$ of the two time periods with different geophone layouts. Before the Geophone #2’s movement, $P_{E}$ similarity showed a rapid increase from 0.36 in the 1st week to 0.81 in the 3rd week. The $P_{E}$ similarity reached about 0.90 in the 4th week (on 5 May 2014), indicating a high reliability of $P_{E}$ results for the seismic analysis. Apart from that, for the 1st week after the movement of Geophone #2 (on 11 June 2014), its $P_{E}$ result still represents high robustness with a value of more than 0.9. This implies that moving Geophone #2 only had minor impacts on the overall $P_{E}$ results.

**Figure 9:**
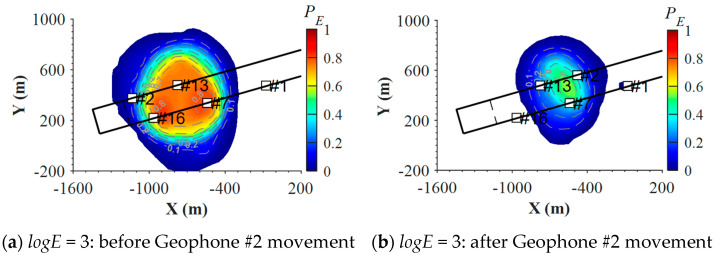

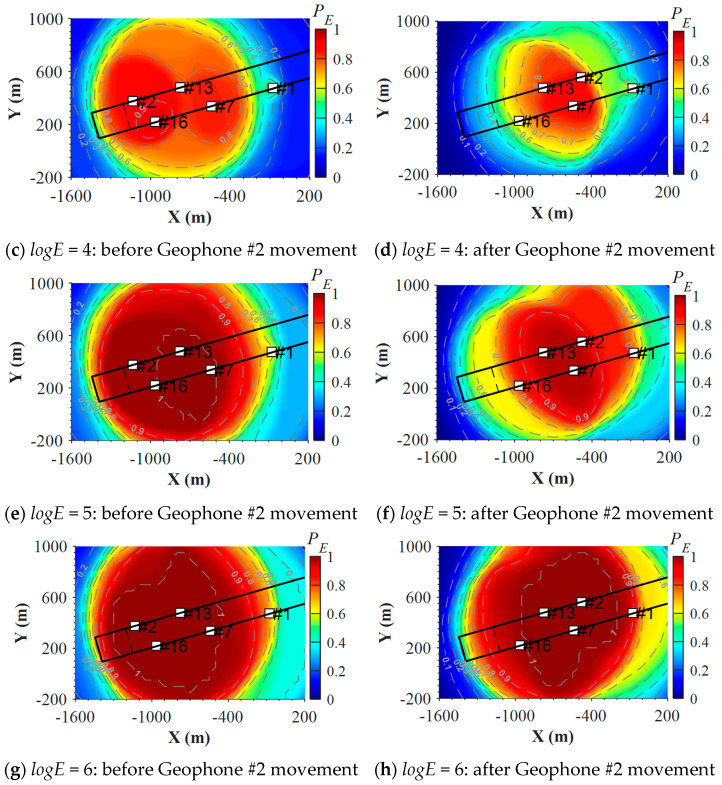
Distribution of detection probability of seismic events with different energies one week before (**left** figures) and after (**right** figures) Geophone #2 had been moved on 5 July 2014 in LW250105. Dashed lines show the position of the longwall face, and white square indicates a geophone.

To investigate the variation characteristics of the detection probability of the seismic monitoring system induced by geophone movement, $P_{E}$ results for the seismic events with *logE* of 3, 4, and 5 one week before and after Geophone #2’s movement on 5 June 2014 were used for analysis, as shown in Figure 9. Figure 9a–d shows that the movement of Geophone #2 significantly weakened the capability of the seismic monitoring system to detect medium-low energy level events. For seismic events with *logE* = 3, the maximum $P_{E}$ was about 0.8 before the geophone movement (Figure 9a), which decreased to less than 0.5 after Geophone #2 was moved (Figure 9b). Similarly, for seismic events with *logE* = 4, high $P_{E}$ zones with values over 0.8 also decreased in size by half after Geophone #2 was moved (see Figure 9c,d). In contrast, geophone movement only causes minor impacts on high-energy level events. Figure 9e,f indicates that after moving Geophone #2, only the zone between Geophone #1 and #7 had a $P_{E}$ reduction for seismic events with *logE* = 5, and the maximum value decreased from 0.9 to 0.8. For seismic events with *logE* = 6, similar $P_{E}$ distributions were presented before and after geophone movement, which only showed limited $P_{E}$ differences outside the geophones enclosed zone (see Figure 9g,h). These results imply that geophone layout modification can lead to significant variations of the detection probability for the medium-low energy level events, but it may only have minor influences on detecting high-energy events.

## 5. Seismic Hazard Forecasting Using Compensated Seismic Data

**Figure 10:**
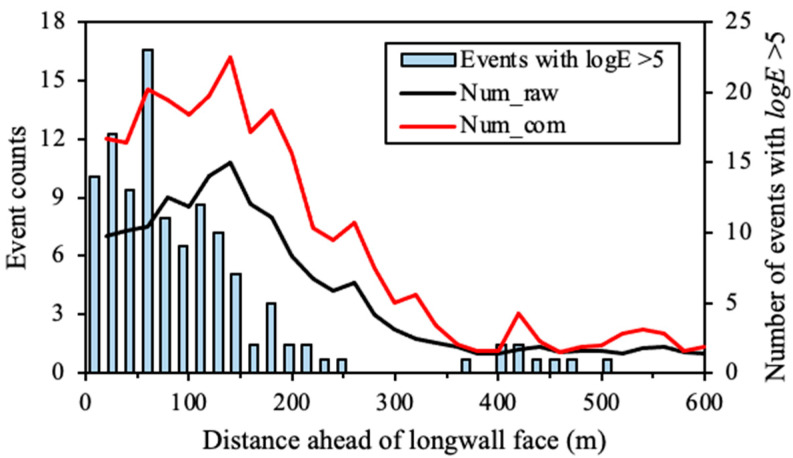
Distribution of average event counts ahead of the longwall face in LW250105 using the compensated seismic data ($Num_{com}$) and raw seismic data ($Num_{raw}$).

**Figure 11:**
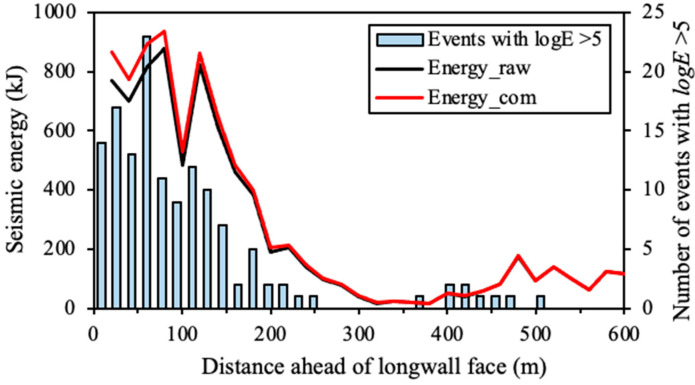
Distribution of average seismic energy ahead of the longwall face in LW250105 using compensated seismic data ($E_{com}$) and raw seismic data ($E_{raw}$).

According to the detection probability characteristics of the seismic monitoring system introduced in Section 4, reliable $P_{E}$ results in LW205015 can be derived after collecting seismic data for at least four weeks. Therefore, the seismic data during the first four weeks in the study period, from 5 April to 5 May 2014, were used to calculate the $P_{E}$ distribution. The compensated seismic data were calculated from 6 May to 1 July 2014. The average event counts and seismic energy ahead of the longwall face were computed using the compensated seismic data, the results of which are shown as red lines in Figure 10 and Figure 11, respectively. For comparison, the average event counts and seismic energy using raw seismic data were also calculated, which are shown as black lines in these figures. Figure 10 shows that the compensated data present much higher average event counts than the raw seismic data within 200 m ahead of the longwall face. The peak of the average event counts is located at 150 m ahead of the longwall face, where 16 and 10 events were presented by using the compensated seismic data and raw seismic data, respectively. This indicates that compensated seismic data can produce about 60% more seismic events than the raw data. For the zone located 60 m ahead of the longwall face, intensive seismic events with *logE* > 5 occurred frequently, indicating an extreme instability of the coal and rock mass. However, the raw seismic data only showed average event counts of seven, which cannot present the actual dynamic failure potential. In contrast, the compensated seismic data showed twice the event counts in the zone (14.5), indicating a high seismic risk. The result shown in Figure 10 supports that the compensated seismic data can better present the instability of the coal and rock mass near the longwall face.

Contrariwise with respect to the event count, the average seismic energy in Figure 11 only shows minor differences between the compensated seismic data and raw seismic data. The compensated seismic data present about 6% higher seismic energy than the raw seismic data in the coal and rock mass located 20–80 m ahead of the longwall face. Nearly identical seismic energy is presented before and after compensating seismic data for zones more than 100 m away from the longwall face. The reason for this phenomenon may be the lower detection probability for medium-low energy seismic events near the longwall face. As shown in Figure 2, the zone within 200 m from the longwall face is not enclosed by geophones. In such a condition, it is more likely for the seismic monitoring system to miss medium-low energy seismic events because of their weak seismic signals. However, even in the unfavourable monitoring zone, it is easier to capture most of the strong seismic events because of their clear seismic signals, which also constitute most of the seismic energy on a scale of logarithm. Therefore, undetected events in the compensated seismic data only constitute a small fraction of all seismic energy released.

**Figure 12:**
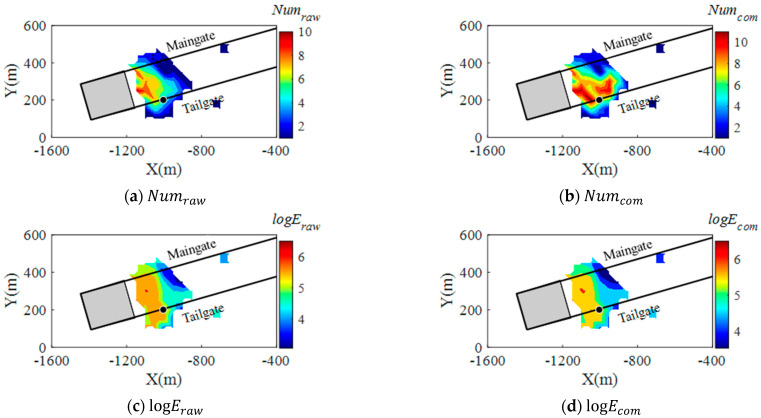
Contours of event counts and seismic energy levels on 31 May 2014 by using raw seismic data (**a**,**c**) and compensated seismic data (**b**,**d**) from 25 May and 31 May 2014. The black dots represent a 100-kJ seismic event that occurred on 1 June 2014, and the grey zone represents the goaf behind the longwall face.

To study the correlation of the compensated seismic data with the seismic risks in LW250105, the daily mapping of event counts and seismic energy, implementing a time window of one week, was conducted during the study period. For each daily mapping result, strong seismic events with *logE* > 5 that occurred at the following day were back analysed. Figure 12b,d and show an example of event counts and seismic energy distribution in LW250105 on 31 May 2014, using the compensated data from 25 May and 31 May 2014. The black dot represents a 100-kJ event that occurred on 1 June 2014. For comparison, the mapping results using raw seismic data are also shown in Figure 12a,c. Figure 12b implies that the compensated seismic data present more intensive seismic activities on the tailgate side of the longwall where a strong seismic event occurred on the following day with $Num_{com}$ = 9. However, the raw seismic data only show a medium degree of event counts in the area where the strong event occurred, with $Num_{raw}$ = 5 (see Figure 12a). For the mapping of seismic energy in the longwall, the $\log E_{raw}$ using the raw seismic data (Figure 12c) and $\log E_{com}$ using the compensated data present nearly identical results. This indicates that limited differences can be obtained for the compensated seismic data in the energy term.

**Figure 13:**
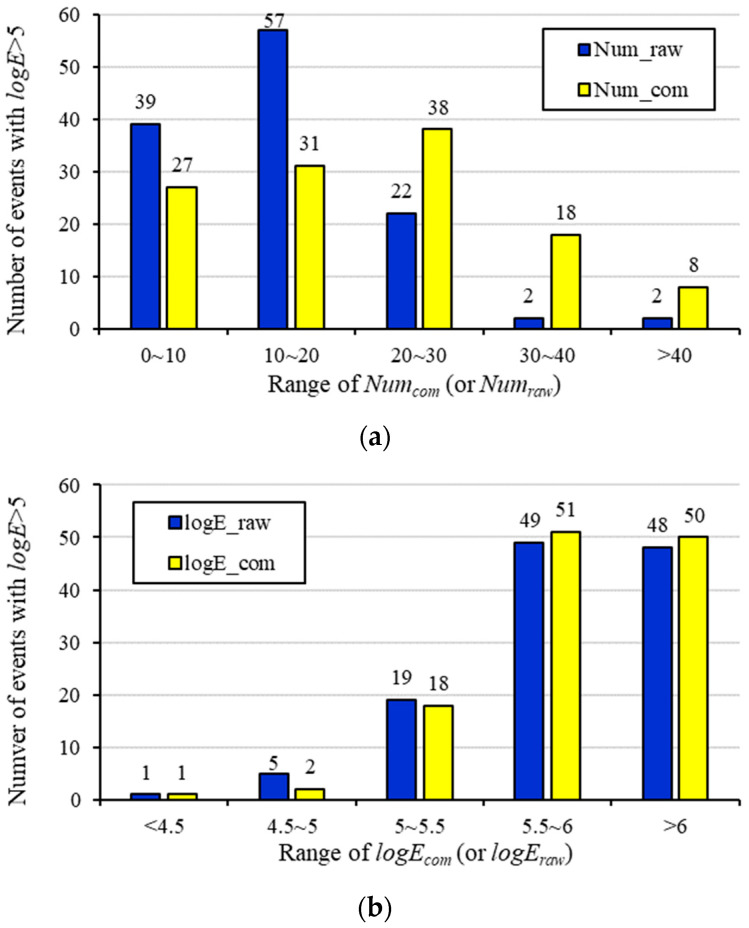
Number of seismic events with logE > 5 in areas with different ranges of (**a**) event counts and (**b**) energies using raw seismic data (blue columns) and compensated seismic data (yellow columns).

From 6 May to 1 July 2014, a total of 122 seismic events with logE > 5 were detected in LW250105, which were used to correlate with $Num_{com}$ and $logE_{com}$, the results of which are shown in Figure 13. For comparison, $Num_{raw}$ and $logE_{raw}$ results that use raw seismic data are also displayed in this figure. Figure 13a shows that more than 50% of the strong seismic events have $Num_{com}$ > 20 based on the compensated seismic data, whereas only about 21% of the events are located in the area where $Num_{raw}$ > 20 when raw seismic data are used. For the significant event counts zone with values >30, 36 seismic events with logE > 5 were detected using $Num_{com}$, which are nine times more than that using $Num_{raw}$. This indicates that the compensated seismic data present a much better correlation with strong seismic events than the raw seismic data, which can be used to improve the accuracy for assessing seismic risks. In contrast, $\log E_{com}$ in Figure 13b only shows limited improvement on the correlation with the strong seismic events compared with $logE_{raw}$. Most seismic events with logE > 5 are located in the area with *logE* > 5.5, which constitute 83% and 80% of the total when using compensated data and raw data, respectively. The reason for this phenomenon may be that seismic events with higher energies commonly have higher detection probabilities. The medium–low energy seismic events that have been missed by the seismic monitoring system only constitute a small fraction of the total released energy, which leads to a similar result between $logE_{com}$ and $logE_{raw}$.

**Figure 14:**
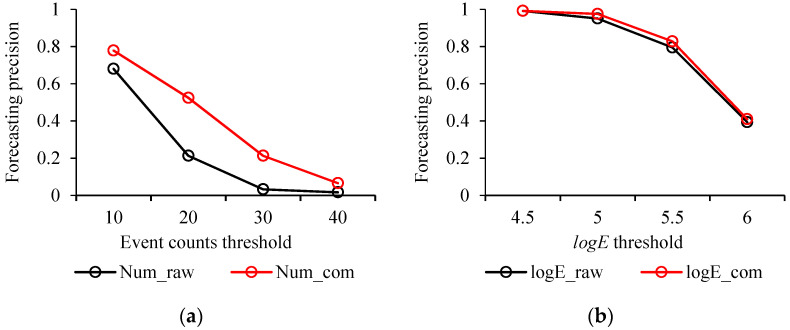

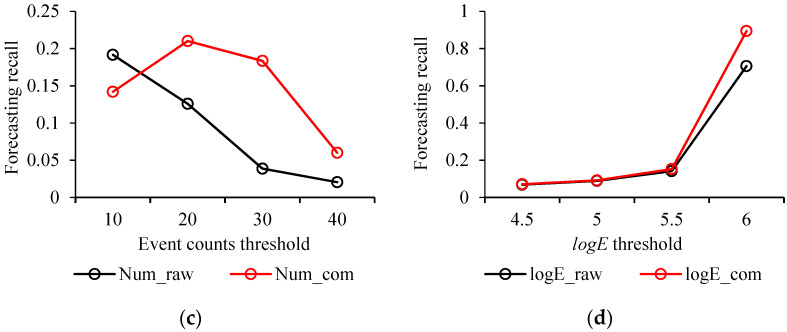
Precision (**a**,**b**) and recall (**c**,**d**) for forecasting logE > 5 events using the raw seismic data and compensated seismic data based on different event counts and energy thresholds.

To evaluate the performance of using compensated seismic data for forecasting strong events, the Confusion Matrix method [32] was adopted to analyse the precision and recall for forecasting logE > 5 events using the raw and compensated seismic data, respectively. The precision and recall are calculated as:

(7) $$\begin{array}{c} \mathrm{precision}=TP/\left(TP+FP\right)\\ \mathrm{recall}=TP/P \end{array}$$

where *TP* denotes true positives, *FP* is false positives, and *P* means total positives. In this study, the precision for strong events forecasting was defined as the ratio of the number of logE > 5 seismic events above the event counts/energy threshold to the total number of logE > 5 seismic events. The recall for the forecasting of strong events was defined as the ratio of the number of grids with logE > 5 seismic events with the event counts/energy above the threshold to the total number of grids with event counts/energy above the threshold. The precision and recall results according to different event counts and energy thresholds are shown in Figure 14. Figure 14a shows that the logE > 5 events forecasting precision using $Num_{raw}$ ($Num_{com}$) decreased with increasing threshold. The forecasting precision using $Num_{com}$ ranged from 0.78 to 0.06 when the threshold changed from 10 to 40, which is higher than that using $Num_{raw}$ with values from 0.68 to 0.02. Compared to $Num_{raw}$ and $Num_{com}$ in Figure 14a, $logE_{raw}$ and $logE_{com}$ in Figure 14b achieved nearly identical precisions for the forecasting of logE > 5 events, which both ranged from 0.99 to 0.4 when the logE threshold changed from 4.5 to 6. In Figure 14c, significant differences are presented in logE > 5 events forecasting recall between using $Num_{raw}$ and $Num_{com}$. At an event counts threshold of 10, $Num_{raw}$ showed a better forecasting recall than $Num_{com}$, which were 0.19 and 0.14, respectively. However, at an event counts threshold of 20, the forecasting recall of $Num_{com}$ increased to 0.21, whereas the forecasting recall of $Num_{raw}$ decreased to 0.12. At an event counts threshold of 30, the recall difference between using $Num_{raw}$ and $Num_{com}$ increased to about 14%, representing 0.04 and 0.18, respectively. The forecasting recall of using $Num_{com}$ significantly decreased to 0.05 at an event counts threshold of 40. In Figure 14d, $\log E_{raw}$ and $\log E_{com}$ present nearly identical forecasting recall values from about 0.07 to 0.14, with the logE threshold ranging from 4.5 to 5.5. When the logE threshold reached 6, $\log E_{com}$ showed a higher forecasting recall than $\log E_{raw}$, which were 0.89 and 0.70, respectively. The results in Figure 14 demonstrate that by analysing event counts and seismic energy using compensated seismic data, the performance of forecasting strong seismic events in mines is further improved. Additionally, from Figure 14, a combination of the event counts and logE thresholds are recommended, which can provide a good method to forecast strong seismic events in mines with an acceptable precision and recall capability. At lower thresholds, event counts and logE both present good precisions, but the recall for logE is relatively low because of the large number of low logE events. At higher thresholds, there is a gain in the recall for logE, but the precision is reduced. Therefore, a good set of thresholds for forecasting strong seismic events could be that event counts equal to 20 and logE equal or better higher than 5.5.

## 6. Conclusions

Incomplete seismic data are the main limitation of the performance of seismic analysing methods used for assessing seismic risks. The *PDE* method provides a means of evaluating the integrity of seismic data by considering the detection probability of the seismic monitoring system in mines, which can be a powerful tool to improve the effectiveness of seismic risk forecasting. Because the detection probability is calculated by using historical raw seismic data, it is essential to determine the seismic data volume required for a reliable result before applying it to the analyses. Therefore, based on three months of seismic data in a burst-prone coal mine, this paper investigated the evolution characteristics of the detection capacities of geophones ($P_{D}$) and the detection probability of the seismic monitoring system ($P_{E}$) during the longwall retreat. The results indicated that a reliable $P_{D}$ result for a geophone commonly requires four weeks of raw seismic data collection, which are equivalent to about 300 seismic events. Geophones that are sensitive to seismic events present significant variations in the $P_{D}$ result in the first three weeks. Reliable $P_{E}$ results can be derived (and used for analyses) after at least four weeks of regular seismic monitoring. In the study case, the seismic monitoring system still presented robust $P_{E}$ results after one geophone was relocated. Based on the $P_{E}$ results, the concept of “compensated seismic data” was proposed to retrieve the seismic events that were missed by the seismic monitoring system. Compared with the raw seismic data, the compensated seismic data showed more intensive seismic activities near the longwall face, indicating instability of the coal and rock mass. The spatial distribution of seismic event counts that use the compensated seismic data also has a higher precision and recall for forecasting future strong seismic events, which can be used to improve the effectiveness for assessing seismic risks.

## Data Availability

The seismic data used in this work are from Huating Coal Mine, and they are confidential.
